# Crystal structure of (2*E*,3*E*)-*N* ^2^,*N* ^3^-bis­(3-ethyl-[1,1′-biphen­yl]-4-yl)butane-2,3-di­imine

**DOI:** 10.1107/S2056989015005071

**Published:** 2015-03-21

**Authors:** Yan Zhao, Jianchao Yuan, Jie Zhao, Shenglan Zhao

**Affiliations:** a Key Laboratory of Eco-Environment-Related Polymer Materials of Ministry of Education, Key Laboratory of Polymer Materials of Gansu Province, College of Chemistry & Chemical Engineering, Northwest Normal University, Lanzhou 730070, People’s Republic of China

**Keywords:** crystal structure, α-di­imine, catalyst, C—H⋯π inter­actions

## Abstract

In the title compound, C_32_H_32_N_2_, synthesized by the con­densation reaction of 2-ethyl-4-phenyl­aniline and 2,3-butane­dione, the conformation about the C=N bonds is *E* and the substituted biphenyl units are *trans* to one another. In the two biphenyl ring systems, the planes of the two rings are inclined to one another by 25.25 (19) and 28.01 (19)°. The planes of the ethyl-substituted benzene rings are inclined to one another by 20.23 (19)° and to the mean plane of the butane-2,3-di­imine unit [maximum deviation = 0.014 (4) Å] by 83.19 (19) and 63.38 (19)°. In the crystal, mol­ecules are linked by C—H⋯π inter­actions, forming sheets lying parallel to (101).

## Related literature   

For literature on α-di­imine palladium and nickel complex catalysts for the polymerization of α-olefins, see: Johnson *et al.* (1995 ▸); Gates *et al.* (2000 ▸). For the crystal structure of a similar compound, see: Chen *et al.* (2014 ▸).
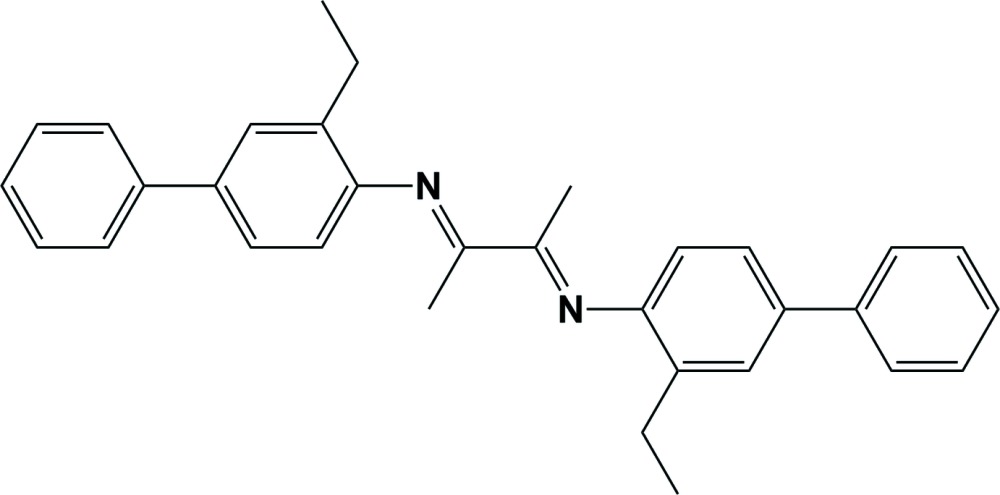


## Experimental   

### Crystal data  

C_32_H_32_N_2_
*M* *_r_* = 444.60
Triclinic, 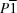

*a* = 9.622 (4) Å
*b* = 9.707 (5) Å
*c* = 14.666 (7) Å
α = 77.288 (5)°
β = 86.934 (4)°
γ = 74.736 (4)°
*V* = 1289.1 (10) Å^3^
*Z* = 2
Mo *K*α radiation
μ = 0.07 mm^−1^
*T* = 296 K
0.28 × 0.26 × 0.25 mm

### Data collection  

Bruker APEXII CCD diffractometer
Absorption correction: multi-scan (*SADABS*; Bruker, 2007 ▸) *T*_min_ = 0.982, *T*_max_ = 0.984
9264 measured reflections
4719 independent reflections
2323 reflections with *I* > 2σ(*I*)
*R*_int_ = 0.042

### Refinement  

*R*[*F* ^2^ > 2σ(*F* ^2^)] = 0.069
*wR*(*F* ^2^) = 0.245
*S* = 1.03
4719 reflections
311 parameters
62 restraints
H-atom parameters constrained
Δρ_max_ = 0.42 e Å^−3^
Δρ_min_ = −0.32 e Å^−3^

### 

Data collection: *APEX2* (Bruker, 2007 ▸); cell refinement: *SAINT* (Bruker, 2007 ▸); data reduction: *SAINT*; program(s) used to solve structure: *SHELXS97* (Sheldrick, 2008 ▸); program(s) used to refine structure: *SHELXL97* (Sheldrick, 2008 ▸); molecular graphics: *SHELXTL* (Sheldrick, 2008 ▸); software used to prepare material for publication: *SHELXTL*.

## Supplementary Material

Crystal structure: contains datablock(s) I, Global. DOI: 10.1107/S2056989015005071/su5091sup1.cif

Structure factors: contains datablock(s) I. DOI: 10.1107/S2056989015005071/su5091Isup2.hkl

Supporting information file. DOI: 10.1107/S2056989015005071/su5091Isup3.cml

**Fig. 1.**
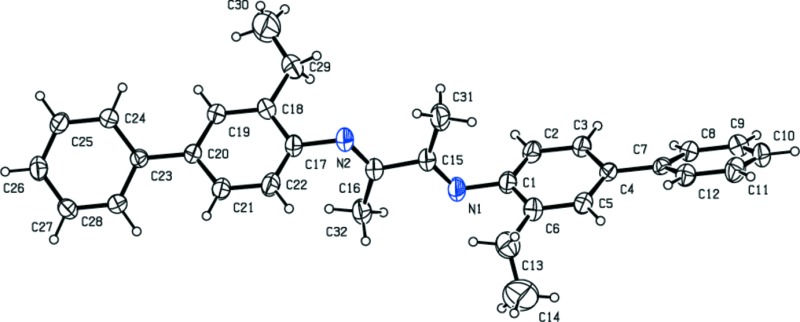
Molecular structure of the title compound, with atom labelling. Displacement ellipsoids are drawn at the 30% probability level.

. DOI: 10.1107/S2056989015005071/su5091fig1.tif
Mol­ecular structure of the title compound, with atom labelling. Displacement ellipsoids are drawn at the 30% probability level.

CCDC reference: 1053619

Additional supporting information:  crystallographic information; 3D view; checkCIF report

## Figures and Tables

**Table 1:** Hydrogen-bond geometry (, ) *Cg*2 and *Cg*4 are the centroids of rings C7-C12 and C23-C28, respectively.

*D*H*A*	*D*H	H*A*	*D* *A*	*D*H*A*
C8H8*Cg*4^i^	0.93	2.83	3.625(5)	144
C11H11*Cg*4^ii^	0.93	2.92	3.639(5)	135
C24H24*Cg*2^iii^	0.93	2.98	3.730(5)	139

Symmetry codes: (i) 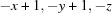; (ii) 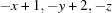; (iii) 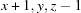.

## supplementary crystallographic information

### S1. Experimental

2-ethyl-4-phenyl­aniline was prepared by dissolving 2-ethyl-4-bromo-aniline (2 mmol, 0.41 g) in PEG-400 (10 ml) containing phenyl­boronic acid (0.293g, 2.4 mmol), K_2_CO_3_ (0.828 g, 0.6 mmol) and PdCl_2_ (50 mg) in a round-bottomed flask and stirred at room temperature for 12 h. On completion of the reaction the solution was purified by column chromatography with ethyl acetate/petroleum ether (v/v = 1:15) as eluent. Pure 2-ethyl-4-phenyl­aniline was obtained as a colourless liquid (yield: 0.385 g, 87%). Formic acid (0.5 ml) was then added to a stirred solution of 2,3-butane­dione (0.043 g, 0.5 mmol) and 2-methyl-4-phenyl­aniline (0.198 g, 1.0 mmol) in ethanol (20 ml). The solid that precipitated was recrystallized from di­chloro­methane/cyclo­hexane (v/v = 30:1), washed with cold cyclo­hexane and dried under vacuum to give the title compound (yield 0.15 g, 84%). Yellow block-like crystals were grown by slow evaporation of a solution of the title compound in a mixture of cyclo­hexane/di­chloro­methane (1:2, v/v).

### S1.1. Refinement

All H atoms were placed in calculated positions and treated as riding: C—H = 0.93 - 0.97 Å with U_iso_(H) = 1.5U_eq_(C) for methyl H atoms and = 1.2U_eq_(C) for other H atoms.

### S2. Comment

In the past few decades, there has been a rapid development of a series of α-diimine palladium and nickel complex catalysts for the polymerization of α-olefins since the discovery of the highly active α-diimine nickel catalysts (Johnson *et al.*, 1995). The study found that nickel metal complex catalysts has a high catalytic activity for ethylene polymerization and high molecular weight polyethylene can be obtained. Palladium metal complex catalysts give highly branched polyethylene and the copolymerization of ethylene and polar monomers have also high catalytically active (Gates *et al.*, 2000). The title compound has been designed to be used as a bidentate ligand for such catalysis.

The molecular structure of the title compound is illustrated in Fig. 1. The molecule is pseudo-centrosymmetric about the central Csp^2^-Csp^2^ bond (C15-C16). The conformation about the C═N bonds (C15═N1 and C16═ N2) is *E* and the substituted biphenyl units are *trans* to one another. In the two biphenyl ring systems the two rings are inclined to one another by 25.25 (19) ° for C1-C6 and C7-C12, and by 28.01 (19) ° for C17-C22 and C23-C28. The ethyl substituted benzene rings (C1-C6 and C17-C22) are inclined to one another by 20.23 (19) ° and to the mean plane of the butane-2,3-diimine unit (maximum deviation = 0.014 (4) Å) by 83.19 (19) and 63.38 (19) °, respectively.

In the crystal, molecules are linked by C-H···π interactions forming sheets lying parallel to (101); see Table 1.

### Crystal data

**Table:**

C_32_H_32_N_2_	*Z* = 2
*M**_r_* = 444.60	*F*(000) = 476
Triclinic, *P*1	*D*_x_ = 1.145 Mg m^−^^3^
Hall symbol: -P 1	Mo *K*α radiation, λ = 0.71073 Å
*a* = 9.622 (4) Å	Cell parameters from 1713 reflections
*b* = 9.707 (5) Å	θ = 2.2–23.0°
*c* = 14.666 (7) Å	µ = 0.07 mm^−^^1^
α = 77.288 (5)°	*T* = 296 K
β = 86.934 (4)°	Block, yellow
γ = 74.736 (4)°	0.28 × 0.26 × 0.25 mm
*V* = 1289.1 (10) Å^3^	

### Data collection

**Table:**

Bruker APEXII CCD diffractometer	4719 independent reflections
Radiation source: fine-focus sealed tube	2323 reflections with *I* > 2σ(*I*)
Graphite monochromator	*R*_int_ = 0.042
φ and ω scans	θ_max_ = 25.5°, θ_min_ = 2.2°
Absorption correction: multi-scan (*SADABS*; Bruker, 2007)	*h* = −10→11
*T*_min_ = 0.982, *T*_max_ = 0.984	*k* = −11→11
9264 measured reflections	*l* = −17→17

### Refinement

**Table:**

Refinement on *F*^2^	Primary atom site location: structure-invariant direct methods
Least-squares matrix: full	Secondary atom site location: difference Fourier map
*R*[*F*^2^ > 2σ(*F*^2^)] = 0.069	Hydrogen site location: inferred from neighbouring sites
*wR*(*F*^2^) = 0.245	H-atom parameters constrained
*S* = 1.03	*w* = 1/[σ^2^(*F*_o_^2^) + (0.0989*P*)^2^ + 0.5812*P*] where *P* = (*F*_o_^2^ + 2*F*_c_^2^)/3
4719 reflections	(Δ/σ)_max_ = 0.001
311 parameters	Δρ_max_ = 0.42 e Å^−^^3^
62 restraints	Δρ_min_ = −0.32 e Å^−^^3^

### Special details

**Table:**

Geometry. All e.s.d.'s (except the e.s.d. in the dihedral angle between two l.s. planes) are estimated using the full covariance matrix. The cell e.s.d.'s are taken into account individually in the estimation of e.s.d.'s in distances, angles and torsion angles; correlations between e.s.d.'s in cell parameters are only used when they are defined by crystal symmetry. An approximate (isotropic) treatment of cell e.s.d.'s is used for estimating e.s.d.'s involving l.s. planes.
Refinement. Refinement of *F*^2^ against ALL reflections. The weighted *R*-factor *wR* and goodness of fit *S* are based on *F*^2^, conventional *R*-factors *R* are based on *F*, with *F* set to zero for negative *F*^2^. The threshold expression of *F*^2^ > σ(*F*^2^) is used only for calculating *R*-factors(gt) *etc*. and is not relevant to the choice of reflections for refinement. *R*-factors based on *F*^2^ are statistically about twice as large as those based on *F*, and *R*- factors based on ALL data will be even larger.

### Fractional atomic coordinates and isotropic or equivalent isotropic displacement parameters (Å^2^)

**Table:**

	*x*	*y*	*z*	*U*_iso_*/*U*_eq_	
C4	0.2795 (3)	0.7460 (4)	0.3800 (2)	0.0475 (9)	
C7	0.2487 (3)	0.7538 (4)	0.4796 (2)	0.0447 (8)	
C24	0.8794 (4)	0.7005 (4)	−0.4998 (2)	0.0547 (9)	
H24	0.9548	0.6738	−0.4571	0.066*	
C23	0.7383 (3)	0.7411 (4)	−0.4689 (2)	0.0447 (8)	
C20	0.7041 (4)	0.7451 (4)	−0.3690 (2)	0.0474 (9)	
N1	0.3646 (4)	0.7226 (4)	0.0967 (2)	0.0691 (10)	
C2	0.4010 (4)	0.6174 (4)	0.2631 (2)	0.0600 (10)	
H2	0.4631	0.5342	0.2484	0.072*	
N2	0.6087 (4)	0.7552 (4)	−0.08534 (19)	0.0677 (9)	
C25	0.9095 (4)	0.6991 (4)	−0.5925 (2)	0.0606 (10)	
H25	1.0046	0.6703	−0.6114	0.073*	
C28	0.6287 (4)	0.7801 (4)	−0.5354 (2)	0.0524 (9)	
H28	0.5332	0.8061	−0.5166	0.063*	
C27	0.6588 (4)	0.7807 (4)	−0.6286 (2)	0.0575 (10)	
H27	0.5841	0.8086	−0.6719	0.069*	
C21	0.5806 (4)	0.8396 (4)	−0.3438 (2)	0.0624 (10)	
H21	0.5183	0.9033	−0.3901	0.075*	
C8	0.2624 (4)	0.6280 (4)	0.5489 (2)	0.0532 (9)	
H8	0.2903	0.5372	0.5328	0.064*	
C3	0.3721 (4)	0.6228 (4)	0.3557 (2)	0.0556 (9)	
H3	0.4149	0.5432	0.4023	0.067*	
C17	0.6367 (4)	0.7479 (4)	−0.1810 (2)	0.0577 (10)	
C12	0.2056 (4)	0.8868 (4)	0.5063 (2)	0.0588 (10)	
H12	0.1960	0.9726	0.4611	0.071*	
C9	0.2352 (4)	0.6355 (5)	0.6412 (2)	0.0638 (11)	
H9	0.2464	0.5499	0.6869	0.077*	
C1	0.3390 (4)	0.7335 (5)	0.1921 (2)	0.0586 (10)	
C15	0.4766 (4)	0.7491 (4)	0.0550 (2)	0.0616 (10)	
C16	0.4957 (4)	0.7297 (4)	−0.0440 (2)	0.0602 (10)	
C22	0.5486 (4)	0.8406 (5)	−0.2514 (2)	0.0705 (12)	
H22	0.4653	0.9056	−0.2363	0.085*	
C11	0.1765 (4)	0.8947 (5)	0.5984 (3)	0.0692 (11)	
H11	0.1466	0.9853	0.6147	0.083*	
C5	0.2159 (4)	0.8603 (4)	0.3076 (2)	0.0571 (10)	
H5	0.1526	0.9429	0.3223	0.069*	
C19	0.7939 (4)	0.6534 (4)	−0.2969 (2)	0.0576 (10)	
H19	0.8780	0.5898	−0.3121	0.069*	
C6	0.2429 (4)	0.8566 (5)	0.2135 (2)	0.0655 (10)	
C18	0.7635 (4)	0.6525 (5)	−0.2029 (2)	0.0655 (11)	
C26	0.7997 (4)	0.7401 (4)	−0.6574 (2)	0.0625 (11)	
H26	0.8204	0.7404	−0.7201	0.075*	
C32	0.3837 (5)	0.6771 (5)	−0.0834 (3)	0.0817 (13)	
H32A	0.2988	0.7562	−0.0990	0.123*	
H32B	0.3602	0.5992	−0.0379	0.123*	
H32C	0.4203	0.6424	−0.1387	0.123*	
C10	0.1917 (4)	0.7683 (5)	0.6659 (3)	0.0724 (12)	
H10	0.1726	0.7731	0.7281	0.087*	
C31	0.5890 (5)	0.7985 (6)	0.0961 (3)	0.1001 (17)	
H31A	0.5484	0.8434	0.1470	0.150*	
H31B	0.6226	0.8680	0.0490	0.150*	
H31C	0.6682	0.7159	0.1186	0.150*	
C29	0.8580 (5)	0.5493 (6)	−0.1247 (3)	0.0996 (15)	
H29A	0.8701	0.6061	−0.0804	0.119*	
H29B	0.8051	0.4804	−0.0930	0.119*	
C13	0.1671 (5)	0.9816 (6)	0.1373 (3)	0.0957 (14)	
H13A	0.1878	1.0705	0.1459	0.115*	
H13B	0.2072	0.9637	0.0776	0.115*	
C30	0.9944 (7)	0.4688 (9)	−0.1444 (4)	0.187 (3)	
H30A	0.9911	0.4376	−0.2018	0.281*	
H30B	1.0250	0.3847	−0.0945	0.281*	
H30C	1.0611	0.5284	−0.1504	0.281*	
C14	0.0125 (7)	1.0059 (9)	0.1331 (5)	0.199 (4)	
H14A	−0.0101	0.9417	0.0981	0.298*	
H14B	−0.0302	1.1055	0.1030	0.298*	
H14C	−0.0248	0.9865	0.1953	0.298*	

### Atomic displacement parameters (Å^2^)

**Table:**

	*U*^11^	*U*^22^	*U*^33^	*U*^12^	*U*^13^	*U*^23^
C4	0.0409 (19)	0.061 (2)	0.0438 (19)	−0.0161 (17)	0.0055 (15)	−0.0145 (17)
C7	0.0345 (18)	0.056 (2)	0.0490 (19)	−0.0165 (16)	0.0058 (14)	−0.0174 (17)
C24	0.047 (2)	0.071 (3)	0.049 (2)	−0.0169 (19)	0.0011 (16)	−0.0179 (18)
C23	0.043 (2)	0.051 (2)	0.0425 (18)	−0.0159 (17)	0.0024 (15)	−0.0124 (15)
C20	0.044 (2)	0.057 (2)	0.0420 (19)	−0.0141 (17)	0.0059 (15)	−0.0122 (16)
N1	0.073 (2)	0.099 (3)	0.0452 (18)	−0.031 (2)	0.0112 (16)	−0.0270 (17)
C2	0.058 (2)	0.069 (3)	0.056 (2)	−0.012 (2)	0.0103 (18)	−0.028 (2)
N2	0.072 (2)	0.091 (3)	0.0439 (17)	−0.0216 (19)	0.0090 (16)	−0.0229 (16)
C25	0.050 (2)	0.079 (3)	0.057 (2)	−0.020 (2)	0.0148 (18)	−0.025 (2)
C28	0.046 (2)	0.063 (2)	0.050 (2)	−0.0159 (18)	0.0015 (16)	−0.0123 (17)
C27	0.059 (2)	0.069 (3)	0.046 (2)	−0.019 (2)	−0.0073 (17)	−0.0099 (17)
C21	0.058 (2)	0.072 (3)	0.046 (2)	−0.002 (2)	0.0077 (17)	−0.0098 (18)
C8	0.051 (2)	0.060 (2)	0.048 (2)	−0.0113 (18)	0.0052 (16)	−0.0161 (17)
C3	0.054 (2)	0.063 (2)	0.051 (2)	−0.0141 (19)	0.0057 (17)	−0.0186 (18)
C17	0.060 (2)	0.075 (3)	0.042 (2)	−0.019 (2)	0.0078 (17)	−0.0194 (18)
C12	0.063 (2)	0.060 (2)	0.057 (2)	−0.021 (2)	0.0075 (18)	−0.0177 (18)
C9	0.064 (3)	0.075 (3)	0.050 (2)	−0.016 (2)	−0.0011 (18)	−0.010 (2)
C1	0.058 (2)	0.080 (3)	0.047 (2)	−0.027 (2)	0.0107 (17)	−0.024 (2)
C15	0.064 (3)	0.081 (3)	0.043 (2)	−0.018 (2)	0.0062 (18)	−0.0205 (19)
C16	0.061 (2)	0.076 (3)	0.044 (2)	−0.015 (2)	0.0052 (18)	−0.0185 (18)
C22	0.065 (3)	0.082 (3)	0.053 (2)	0.001 (2)	0.0136 (19)	−0.017 (2)
C11	0.078 (3)	0.071 (3)	0.066 (3)	−0.021 (2)	0.013 (2)	−0.032 (2)
C5	0.055 (2)	0.064 (2)	0.052 (2)	−0.0144 (19)	0.0105 (17)	−0.0154 (18)
C19	0.050 (2)	0.073 (3)	0.048 (2)	−0.0078 (19)	0.0029 (16)	−0.0181 (18)
C6	0.067 (2)	0.079 (3)	0.049 (2)	−0.021 (2)	0.0078 (18)	−0.0092 (18)
C18	0.059 (2)	0.093 (3)	0.0417 (19)	−0.014 (2)	−0.0026 (16)	−0.0144 (19)
C26	0.075 (3)	0.076 (3)	0.045 (2)	−0.029 (2)	0.0101 (19)	−0.0212 (19)
C32	0.084 (3)	0.120 (4)	0.053 (2)	−0.037 (3)	0.008 (2)	−0.033 (2)
C10	0.074 (3)	0.102 (4)	0.049 (2)	−0.024 (3)	0.008 (2)	−0.032 (2)
C31	0.092 (3)	0.176 (5)	0.060 (3)	−0.065 (4)	0.016 (2)	−0.050 (3)
C29	0.085 (3)	0.142 (4)	0.053 (2)	0.001 (3)	−0.009 (2)	−0.017 (2)
C13	0.095 (3)	0.104 (3)	0.070 (3)	−0.011 (3)	0.000 (2)	0.002 (2)
C30	0.137 (5)	0.249 (7)	0.089 (4)	0.063 (5)	−0.009 (4)	0.009 (4)
C14	0.137 (5)	0.234 (7)	0.137 (5)	0.028 (6)	−0.017 (4)	0.057 (5)

### Geometric parameters (Å, º)

**Table:**

C4—C5	1.388 (5)	C9—H9	0.9300
C4—C3	1.395 (5)	C1—C6	1.391 (5)
C4—C7	1.489 (4)	C15—C31	1.494 (6)
C7—C12	1.384 (5)	C15—C16	1.501 (5)
C7—C8	1.387 (5)	C16—C32	1.497 (5)
C24—C25	1.377 (4)	C22—H22	0.9300
C24—C23	1.391 (4)	C11—C10	1.377 (6)
C24—H24	0.9300	C11—H11	0.9300
C23—C28	1.394 (4)	C5—C6	1.396 (5)
C23—C20	1.491 (4)	C5—H5	0.9300
C20—C21	1.386 (5)	C19—C18	1.392 (5)
C20—C19	1.392 (5)	C19—H19	0.9300
N1—C15	1.269 (4)	C6—C13	1.510 (6)
N1—C1	1.430 (4)	C18—C29	1.506 (5)
C2—C3	1.380 (4)	C26—H26	0.9300
C2—C1	1.381 (5)	C32—H32A	0.9600
C2—H2	0.9300	C32—H32B	0.9600
N2—C16	1.272 (4)	C32—H32C	0.9600
N2—C17	1.428 (4)	C10—H10	0.9300
C25—C26	1.379 (5)	C31—H31A	0.9600
C25—H25	0.9300	C31—H31B	0.9600
C28—C27	1.380 (4)	C31—H31C	0.9600
C28—H28	0.9300	C29—C30	1.391 (6)
C27—C26	1.379 (5)	C29—H29A	0.9700
C27—H27	0.9300	C29—H29B	0.9700
C21—C22	1.375 (5)	C13—C14	1.446 (7)
C21—H21	0.9300	C13—H13A	0.9700
C8—C9	1.379 (5)	C13—H13B	0.9700
C8—H8	0.9300	C30—H30A	0.9600
C3—H3	0.9300	C30—H30B	0.9600
C17—C22	1.370 (5)	C30—H30C	0.9600
C17—C18	1.394 (5)	C14—H14A	0.9600
C12—C11	1.379 (5)	C14—H14B	0.9600
C12—H12	0.9300	C14—H14C	0.9600
C9—C10	1.369 (6)		
			
C5—C4—C3	117.2 (3)	C21—C22—H22	119.3
C5—C4—C7	121.4 (3)	C10—C11—C12	119.8 (4)
C3—C4—C7	121.3 (3)	C10—C11—H11	120.1
C12—C7—C8	117.7 (3)	C12—C11—H11	120.1
C12—C7—C4	121.1 (3)	C4—C5—C6	122.8 (4)
C8—C7—C4	121.2 (3)	C4—C5—H5	118.6
C25—C24—C23	121.2 (3)	C6—C5—H5	118.6
C25—C24—H24	119.4	C20—C19—C18	123.0 (3)
C23—C24—H24	119.4	C20—C19—H19	118.5
C24—C23—C28	117.4 (3)	C18—C19—H19	118.5
C24—C23—C20	121.8 (3)	C1—C6—C5	118.2 (4)
C28—C23—C20	120.8 (3)	C1—C6—C13	121.0 (4)
C21—C20—C19	117.0 (3)	C5—C6—C13	120.7 (4)
C21—C20—C23	121.4 (3)	C19—C18—C17	117.9 (3)
C19—C20—C23	121.7 (3)	C19—C18—C29	122.9 (4)
C15—N1—C1	120.7 (3)	C17—C18—C29	119.1 (3)
C3—C2—C1	121.0 (4)	C25—C26—C27	119.4 (3)
C3—C2—H2	119.5	C25—C26—H26	120.3
C1—C2—H2	119.5	C27—C26—H26	120.3
C16—N2—C17	122.1 (3)	C16—C32—H32A	109.5
C24—C25—C26	120.5 (3)	C16—C32—H32B	109.5
C24—C25—H25	119.8	H32A—C32—H32B	109.5
C26—C25—H25	119.8	C16—C32—H32C	109.5
C27—C28—C23	121.4 (3)	H32A—C32—H32C	109.5
C27—C28—H28	119.3	H32B—C32—H32C	109.5
C23—C28—H28	119.3	C9—C10—C11	119.8 (4)
C26—C27—C28	120.1 (3)	C9—C10—H10	120.1
C26—C27—H27	120.0	C11—C10—H10	120.1
C28—C27—H27	120.0	C15—C31—H31A	109.5
C22—C21—C20	121.0 (4)	C15—C31—H31B	109.5
C22—C21—H21	119.5	H31A—C31—H31B	109.5
C20—C21—H21	119.5	C15—C31—H31C	109.5
C9—C8—C7	121.1 (4)	H31A—C31—H31C	109.5
C9—C8—H8	119.5	H31B—C31—H31C	109.5
C7—C8—H8	119.5	C30—C29—C18	119.9 (4)
C2—C3—C4	120.8 (3)	C30—C29—H29A	107.3
C2—C3—H3	119.6	C18—C29—H29A	107.3
C4—C3—H3	119.6	C30—C29—H29B	107.3
C22—C17—C18	119.8 (3)	C18—C29—H29B	107.3
C22—C17—N2	121.5 (3)	H29A—C29—H29B	106.9
C18—C17—N2	118.5 (3)	C14—C13—C6	115.4 (4)
C11—C12—C7	121.4 (4)	C14—C13—H13A	108.4
C11—C12—H12	119.3	C6—C13—H13A	108.4
C7—C12—H12	119.3	C14—C13—H13B	108.4
C10—C9—C8	120.3 (4)	C6—C13—H13B	108.4
C10—C9—H9	119.9	H13A—C13—H13B	107.5
C8—C9—H9	119.9	C29—C30—H30A	109.5
C2—C1—C6	119.8 (3)	C29—C30—H30B	109.5
C2—C1—N1	120.1 (4)	H30A—C30—H30B	109.5
C6—C1—N1	119.9 (4)	C29—C30—H30C	109.5
N1—C15—C31	125.4 (3)	H30A—C30—H30C	109.5
N1—C15—C16	116.5 (4)	H30B—C30—H30C	109.5
C31—C15—C16	118.1 (3)	C13—C14—H14A	109.5
N2—C16—C32	126.3 (3)	C13—C14—H14B	109.5
N2—C16—C15	116.3 (4)	H14A—C14—H14B	109.5
C32—C16—C15	117.3 (3)	C13—C14—H14C	109.5
C17—C22—C21	121.4 (4)	H14A—C14—H14C	109.5
C17—C22—H22	119.3	H14B—C14—H14C	109.5
			
C5—C4—C7—C12	−25.7 (5)	C17—N2—C16—C15	−176.9 (4)
C3—C4—C7—C12	155.0 (3)	N1—C15—C16—N2	−179.4 (4)
C5—C4—C7—C8	154.3 (3)	C31—C15—C16—N2	1.0 (6)
C3—C4—C7—C8	−24.9 (5)	N1—C15—C16—C32	−1.7 (6)
C25—C24—C23—C28	0.1 (5)	C31—C15—C16—C32	178.8 (4)
C25—C24—C23—C20	−179.2 (3)	C18—C17—C22—C21	1.6 (6)
C24—C23—C20—C21	151.7 (4)	N2—C17—C22—C21	175.9 (4)
C28—C23—C20—C21	−27.6 (5)	C20—C21—C22—C17	−0.5 (6)
C24—C23—C20—C19	−28.9 (5)	C7—C12—C11—C10	0.7 (6)
C28—C23—C20—C19	151.8 (3)	C3—C4—C5—C6	−1.0 (5)
C23—C24—C25—C26	0.9 (6)	C7—C4—C5—C6	179.7 (3)
C24—C23—C28—C27	−1.1 (5)	C21—C20—C19—C18	0.7 (6)
C20—C23—C28—C27	178.2 (3)	C23—C20—C19—C18	−178.7 (3)
C23—C28—C27—C26	1.1 (5)	C2—C1—C6—C5	2.1 (6)
C19—C20—C21—C22	−0.6 (6)	N1—C1—C6—C5	177.1 (3)
C23—C20—C21—C22	178.8 (3)	C2—C1—C6—C13	−176.5 (4)
C12—C7—C8—C9	−0.6 (5)	N1—C1—C6—C13	−1.5 (6)
C4—C7—C8—C9	179.3 (3)	C4—C5—C6—C1	−0.8 (6)
C1—C2—C3—C4	−0.1 (6)	C4—C5—C6—C13	177.9 (4)
C5—C4—C3—C2	1.4 (5)	C20—C19—C18—C17	0.3 (6)
C7—C4—C3—C2	−179.3 (3)	C20—C19—C18—C29	177.7 (4)
C16—N2—C17—C22	63.3 (6)	C22—C17—C18—C19	−1.4 (6)
C16—N2—C17—C18	−122.3 (4)	N2—C17—C18—C19	−175.9 (3)
C8—C7—C12—C11	−0.3 (5)	C22—C17—C18—C29	−178.9 (4)
C4—C7—C12—C11	179.8 (3)	N2—C17—C18—C29	6.6 (6)
C7—C8—C9—C10	1.0 (6)	C24—C25—C26—C27	−0.9 (6)
C3—C2—C1—C6	−1.7 (6)	C28—C27—C26—C25	0.0 (6)
C3—C2—C1—N1	−176.7 (3)	C8—C9—C10—C11	−0.6 (6)
C15—N1—C1—C2	−84.0 (5)	C12—C11—C10—C9	−0.2 (6)
C15—N1—C1—C6	100.9 (5)	C19—C18—C29—C30	12.0 (9)
C1—N1—C15—C31	−2.4 (7)	C17—C18—C29—C30	−170.6 (6)
C1—N1—C15—C16	178.0 (4)	C1—C6—C13—C14	114.5 (6)
C17—N2—C16—C32	5.6 (6)	C5—C6—C13—C14	−64.1 (7)

### Hydrogen-bond geometry (Å, º)

Cg2 and Cg4 are the centroids of rings C7-C12 and C23-C28, respectively.

**Table:**

*D*—H···*A*	*D*—H	H···*A*	*D*···*A*	*D*—H···*A*
C8—H8···*Cg*4^i^	0.93	2.83	3.625 (5)	144
C11—H11···*Cg*4^ii^	0.93	2.92	3.639 (5)	135
C24—H24···*Cg*2^iii^	0.93	2.98	3.730 (5)	139

Symmetry codes: (i) −*x*+1, −*y*+1, −*z*; (ii) −*x*+1, −*y*+2, −*z*; (iii) *x*+1, *y*, *z*−1.
