# Supplementary figures and images for: Crystal structure of (2E,3E)-N 2,N 3-bis­(3-ethyl-[1,1′-biphen­yl]-4-yl)butane-2,3-di­imine
Source: Acta Crystallogr E Crystallogr Commun. 2015 Mar 21;71(Pt 4):o251–2. doi: 10.1107/S2056989015005071 (PMC4438819; doi:10.1107/S2056989015005071)

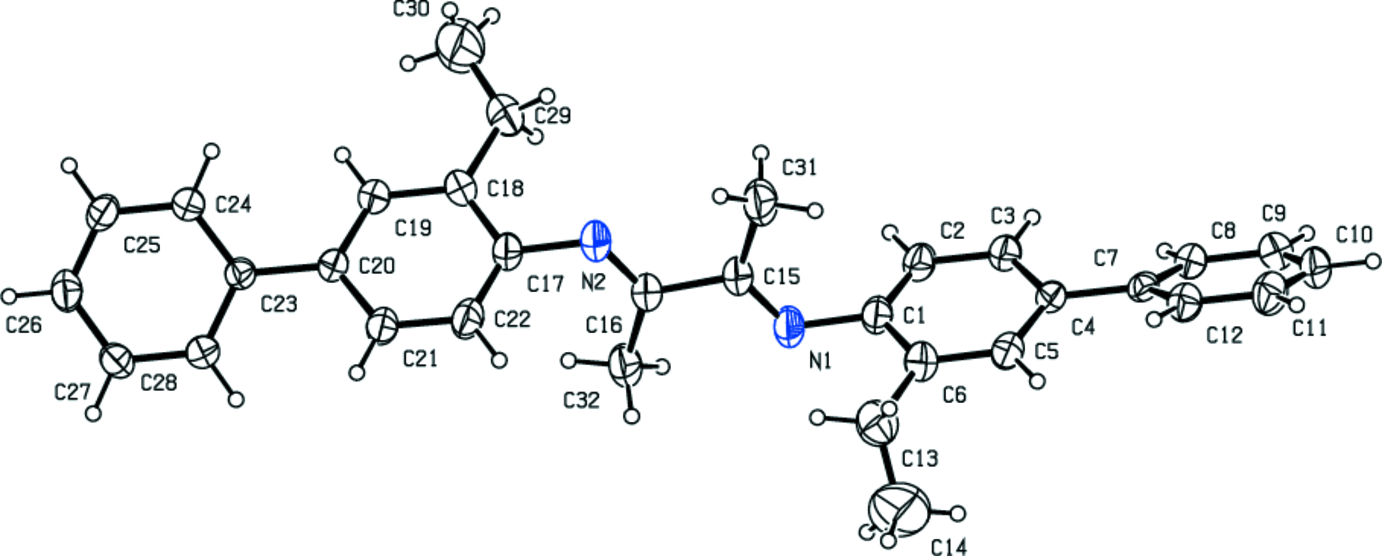

Supplement: Supplementary file 4 [file e-71-0o251-fig1.tif]
